# Intervention to Increase HIV Testing Among Substance-Using Young Men Who Have Sex With Men: Protocol for a Randomized Controlled Trial

**DOI:** 10.2196/resprot.9414

**Published:** 2018-04-30

**Authors:** Rob Stephenson, Erin E Bonar, Adam Carrico, Alexis Hunter, Daniel Connochie, Rebecca Himmelstein, Jose Bauermeister

**Affiliations:** ^1^ Center for Sexuality & Health Disparities University of Michigan Ann Arbor, MI United States; ^2^ Addiction Center Department of Psychiatry University of Michigan Ann Arbor, MI United States; ^3^ School of Public Health University of Miami Miami, FL United States; ^4^ Program on Sexuality, Technology & Action Research School of Nursing University of Pennsylvania Philadelphia, PA United States

**Keywords:** HIV, men who have sex with men, drug abuse, substance use disorders, motivational interviewing

## Abstract

**Background:**

Young men who have sex with men (YMSM) and transgender people in the Detroit Metro Area are the only risk group for whom the incidence of HIV and sexually transmitted infections (STI) has increased since 2000, with HIV incidence nearly doubling among youth. Substance use (including alcohol), which is relatively frequent among YMSM and transgender people, creates barriers to the optimal delivery of HIV prevention and care services. Standard HIV counseling, testing, and referral (CTR) is limited in providing strategies to identify and address substance use. Hence, in its current form, CTR may not be serving the prevention needs of substance-using YMSM and transgender people. Brief counseling interventions, grounded in principles of motivational interviewing, may offer a mechanism to meet the HIV prevention and care needs of substance-using YMSM and transgender people.

**Objective:**

This prospective, 4-arm, factorial randomized controlled trial aims to examine the efficacy of an motivational interviewing–based substance use brief intervention (SUBI) on participants’ substance use and engagement in HIV prevention.

**Methods:**

The research implements a prospective randomized controlled trial (Project Swerve) of 600 YMSM and transgender people recruited both online and in person. Eligibility criteria include participants who (1) are between the ages of 15 to 29 years, (2) live in the Detroit Metro Area, (3) self-identify as a man or transgender man or woman, (4) have had sexual contact with a man in the 6 months before enrollment, (5) self-report binge drinking or any substance use in the 3 months before enrollment, and (6) self-report an unknown or negative HIV status upon enrollment. Participants are randomized to receive, 3-months apart starting at baseline, 2 individual sessions. Sessions are CTR-only, SUBI-only, CTR followed by SUBI, or SUBI followed by CTR.

**Results:**

Project Swerve was launched in April 2017 and enrollment is ongoing.

**Conclusions:**

Incorporating a SUBI that utilizes the principles of motivational interviewing into HIV CTR provides an opportunity to tailor counseling services for YMSM and transgender people to address additional client barriers to HIV and STI testing.

**Trial Registration:**

ClinicalTrials.gov NCT02945436; http://clinicaltrials.gov/ct2/show/NCT02945436 (Archived by WebCite at http://www.webcitation.org/6yFyOK57w)

## Introduction

### Background

Young men who have sex with men (YMSM) and transgender people (TG; herein collectively referred to as YMSMTG) are at heightened risk for HIV and other sexually transmitted infections (STI) [1,2]. The 3 major cities in the Detroit Metro Area (DMA)—Detroit, Flint, and Ann Arbor—are represented in the top 5 Michigan counties with greatest increases of new HIV, chlamydia, gonorrhea, and syphilis infections [1]. Consistent with the national epidemic, YMSMTG in the DMA are the only risk groups for whom HIV and STI incidence has increased since 2000, with HIV incidence among YMSM between the ages of 13 and 25 years nearly doubling [1,2]. YMSM accounted for 72% of new HIV infections and over 80% of new syphilis diagnoses among people aged 13-24 years. Over 75% of gonorrhea-HIV coinfections were among YMSM [1,3].

The Centers for Disease Control and Prevention (CDC) have recommended that HIV and STI testing be repeated frequently (3-6 month intervals) for high-risk YMSMTG (ie, having multiple or anonymous partners with whom they have condomless anal intercourse [CAI] and who report engaging in illicit drug use) [2]. Recent recommendations from a CDC working group on HIV testing for men who have sex with men (MSM) suggest that clinicians can consider the benefits of offering more frequent screening (eg, once every 3 or 6 months) to individual MSM at increased risk for acquiring HIV infection, weighing their patients’ individual risk factors, local HIV epidemiology, and local testing policies [2]. Consistent with national trends, YMSM living in the DMA report low adherence to these testing guidelines; data are scant on whether TG in this context are adhering to testing guidelines. In 3 prior studies conducted between 2011 and 2014 with YMSM in this community [4-6], a large proportion of YMSM (15%-36%) had never tested for HIV. Among those who did test for HIV, over 65% reported that they had not tested in the past 12 months. YMSM living with HIV also account for the largest drop-off across the HIV/AIDS continuum of care in the DMA, particularly if they are racial or ethnic minorities and live in neighborhoods characterized by socioeconomic disadvantage [1].

Changes in the use of alcohol, tobacco, and other drugs (ATOD) during adolescence and young adulthood are developmentally noteworthy because they can have short- and long-term consequences that affect one’s adult life trajectory including HIV/STI acquisition [7], development of substance use disorders (SUDs), and disruptions in school and job performance [8-14]. Alcohol and marijuana are the most common substances used by youth. National Survey on Drug Use and Health [15] data for the DMA demonstrated that 12.1% of 18- to 25-year-olds needed, but did not receive, treatment for alcohol use and 6.8% of 18- to 25-year-olds needed, but did not receive, treatment for drug use. Given the known synergy between AOD use and HIV risk among YMSMTG [16-21], there is a need to develop HIV prevention interventions that also recognize and tackle issues of substance use [22].

AIDS Service Organizations (ASOs) often serve and are sensitive to the HIV-related needs of underserved YMSMTG. Delivery of HIV services through ASOs has been an efficient rollout mechanism because they reach and affect large numbers of people efficiently; create and establish grassroots policies and procedures that maximize the adoption and diffusion of interventions while considering the community’s social context; increase program sustainability and advocacy; and incorporate the needs of specific communities into tailored services. At present, however, HIV test counselors situated in ASOs are not trained to comprehensively and systematically screen for and address ATOD use in counseling, testing, and referral (CTR) sessions—the routine procedure used to test for HIV. Preliminary data from our community partners indicate that lack of AOD screening and counseling within CTR is a missed opportunity. The authors of this study [23] and others [7,24,25] have also documented that HIV-positive persons with problematic patterns of alcohol and stimulant use experience difficulties with HIV disease management and display elevated HIV viral load, demonstrating a need for reducing substance use early to avoid complicating disease management.

Consistent with the National HIV/AIDS Strategy’s call [26] to reduce new HIV infections by intensifying prevention efforts in highly impacted communities and increasing rates of routine HIV testing, this protocol outlines an intervention that targets high-risk YMSMTG by including a substance use brief intervention (SUBI) as part of CTR. The intervention builds on prior SUBI research [27-34] and also meets the recommendations of the CDC working group on HIV testing among MSM that recommended more frequent testing among high-risk groups: substance using YMSMTG are clearly at an elevated risk of HIV acquisition and currently underutilize HIV prevention services. Using a consensus approach to conceptualize health behavior change, the model guiding our intervention [35-38] synthesizes social cognitive theories [39], along with the trans-theoretical model of change [40,41] and self-determination theory [42,43]. These theories emphasize social cognitive factors that impact behavior change and have informed ATOD and HIV interventions [12,44-46]. Motivational interviewing (MI) [36], the primary approach used to deliver SUBI, is consistent with these theories [47], focusing on resolving ambivalence about problem behaviors, increasing self-efficacy for change, and enhancing motivation moving toward action [48]. This protocol describes the methods for the testing of the intervention below.

### Objective

The prospective, 4-arm factorial randomized controlled trial (RCT) aims to examine the efficacy of Project Swerve, an MI-based SUBI (intervention) compared with the current standard of care CTR (control) on participants’ substance use and engagement in HIV prevention.

## Methods

### Trial Registration, Ethics, Consent, and Institutional Board Approval

The research and ethics presented in this study has been reviewed and approved by the University of Michigan Institutional Review Board (HUM00105125), in addition to the Data Safety Monitoring Board. The study is also registered on ClinicalTrials.gov (NCT02945436).

### Trial Design

The research activities involve a 4-arm factorial RCT of approximately 600 AOD-using YMSMTG aged 15-29 years in the DMA. We will follow participants for 18 months, with follow-up assessments collected every 3 months.

The intervention comprises 2 visits separated by 3 months. Participants are randomized to receive either CTR (control) or a SUBI-adapted version of CTR (referred to as SUBI; intervention) in each visit. To examine how the sequencing and dosing of interventions impacts efficacy, we randomize at baseline into a factorial RCT. The control arm will receive CTR-only at both study visits 1 and 2. Experimental arm 1 (CTR+SUBI) will receive CTR at visit 1 and SUBI at visit 2. Experimental arm 2 (SUBI+CTR) will receive SUBI at visit 1 and CTR at visit 2. Experimental arm 3 (SUBI+SUBI) will receive the intervention condition at visits 1 and 2. Individuals who test HIV-positive at study visits 1 or 2 will receive case management and linkage to care, as offered routinely by each ASO where study activities take place.

This 4-arm factorial randomized design will help answer 3 important questions: (1) what is the impact of adapting current CTR to include SUBI on HIV engagement in care and sexual- and substance-related risk-taking behaviors among high-risk YMSMTG; (2) what combination of services (CTR-only, CTR+SUBI, SUBI+CTR, SUBI+SUBI) has the greatest impact on engagement in HIV prevention (where engagement in care is defined as routine HIV testing for seronegative YMSMTG and linkage and retention in care for seropositive YMSMTG); and (3) if effective, what are the costs of delivering SUBI compared with those of delivering CTR?

### Participants

Eligible participants are: (1) between 15 and 29 years old, (2) identify as a cisgender man or as transgender man or woman, (3) have had sexual contact with a man (oral or anal) in the last 6 months, (4) live in the DMA, (5) have unknown or negative HIV status, (6) and report binge drinking or using any illicit substance or nonmedical use of prescription drugs in the prior 3 months. The ATOD eligibility criteria are measured using the Alcohol, Smoking and Substance Involvement Screening Test (ASSIST) [49] to assess frequency of AOD use in the prior 3 months.

### Recruitment

Participants are recruited using Web-based advertisements on social media websites (eg, Facebook, Grindr), which will be tailored to target YMSMTG in the DMA. Recruitment will also occur in person at local venues and community outreach events in the region.

### Screening

Eligible participants will be invited for their baseline visit at the ASO closest to them: offices are available in Ann Arbor, Ypsilanti, Detroit, and Flint, allowing access by participants from across the DMA. Participants who present for participation in the trial will have already set up a study account online and completed an online baseline survey with informed consent given online. When they arrive for their first visit, they will be reconsented verbally and offered a physical copy of the consent form. A study counselor will be available to answer any questions that the participants may have about the study before they decide to participate. Participants who do not consent to participate in the trial will be offered free HIV testing and counseling. Resources will be readily available for providing tools to avoid ATOD-related risks (eg, reducing ATOD use or consequences), and referrals to community resources as needed (eg, psychosocial services, leisure activities not involving substance use).

### Randomization

Participants will be randomly assigned to 1 of the 4 conditions using a 1:1:1:1 treatment allocation. The treatment assignments will be generated with the use of a pseudo-random-number generator with permutated blocks that will be used to ensure balance between the numbers of YMSMTG assigned to each treatment. Assignments to the control or intervention arms will use concealment of allocation techniques designed to minimize assignment bias including generating, in advance, the sequence of assignment in sealed envelopes, which will be opened by the counselor at the time of randomization. When YMSM are randomized to receive CTR-only at a visit, they will receive standard CTR (30 min). YMSM randomized to SUBI at a study visit will receive the CTR that has been adapted to include the SUBI. On average, both conditions will last approximately 30 min.

### Incentives

Participants will receive US $25 at each ASO visit and US $30 for each follow-up, making the total potential incentives (if all assessment visits are completed) US $200 per participant.

### Intervention

Study interventionists will be trained to deliver SUBI and will offer YMSMTG the opportunity to explore and strengthen motivations for changing their ATOD use during session. Interventionists are trained in CTR and typically have a Master’s degree in Public Health, Social Work, or a health-related discipline. The SUBI intervention consists of 2 components. Similar in style to other MI-based brief interventions for substance use and related risk behaviors [50-54], Component 1 focuses on employing MI to explore substance use (illicit drugs, misuse of prescription drugs, alcohol) and co-occurring sexual risk-taking with cultural and developmental tailoring for YMSMTG. There are 7 steps to Component 1 (see Table 1). To maintain the MI spirit, participants are asked for permission to begin the session and also when transitioning through different steps of each component.

**Table 1:** Steps taken during Component 1 of the Swerve intervention compared with standard counseling, testing, and referral (CTR) steps.

Steps	Swerve intervention (SUBI^a^) Component 1: Alcohol, drugs, and sex	CTR: HIV prevention counseling
1	Rapport-building, exploring participants’ strengths and near-term goals	Introduce and orient the participant/client to the session and conduct of HIV test
2	Review of alcohol and substance use and conduct of HIV test	Identify risk behaviors and circumstances
3	Psychoeducation about alcohol/drugs and HIV risk	Identify safer goal behavior
4	Explore benefits to reducing substance use/harm reduction	Identify action steps
5	Build commitment to change	Provide referrals and support
6	Summary of Steps 1-6	Summarize and close
7	Explore possible reactions to HIV test results	—

^a^SUBI: substance use brief intervention.

In Step 1, counselors focus on MI-based engagement strategies to explore areas of strength and aspirations that the participant holds. Affirming these areas allows counselors to build rapport with participants and begin to uncover potential sources of motivation to change risky behaviors.

Step 2 invokes the MI process of focusing by reviewing participants’ recent substance use. Counselors explore participants’ frequency of current substance use, their motivations for use, and elicit any consequences to using substances. Possible links between substance use and risky sexual behaviors are examined by querying the potential role of substance use in having sex or hooking up and use of condoms. Counselors are trained to listen for, elaborate on, and evoke change talk as it begins to occur in Step 2 and throughout the remainder of the session. In Step 2, the counselor conducts the rapid HIV test.

In Step 3, counselors provide basic psychoeducation about how substance use and/or risky sex can impact risk for HIV infection, tailored to the participant’s own high-risk behaviors.

Step 4 seeks to elicit from participants any potential benefits to changing their substance use (eg, reducing use, ceasing use, or employing harm reduction), with a specific emphasis on how changing use can impact the risk for HIV transmission.

In Step 5, counselors reflect on participants’ perceived benefits and assess the importance of and readiness to change using the visual of a ruler in order to elicit their current stage of change. If participants are interested in changing, the counselor uses evocative questions to elicit a potential first step; for those not interested in making changes currently, the counselor elicits participants’ views on what might prompt them to consider a change in the future.

In Step 6, the counselor provides a strategic summary of what was discussed during Component 1. Here, counselors are beginning to transition into disclosing the HIV results from the test that was conducted in Step 2 and it is important for participants to think about what was discussed in each step as a whole.

Finally, in Step 7, counselors elicit and reflect how participants would react to a positive (reactive) or negative (nonreactive) result before disclosing the HIV results.

Component 2 varies based on the HIV test results (see Table 2), with the focus across intervention arms including either risk reduction counseling for HIV-negative participants or linkage and retention to HIV care among newly HIV-diagnosed individuals. Throughout Component 2, counselors remain grounded in the MI spirit and use MI skills to engage the participant in a collaborative conversation.

If participants’ results are nonreactive, repeat HIV testing and pre-exposure prophylaxis (PrEP) are the focal points of Steps 8 through 12. In Step 8, counselors elicit and reflect participants’ responses to receiving a nonreactive result.

Step 9 explores the benefits of repeat testing. Counselors discuss the window period, recommendation for testing every 3-6 months, and how participants feel about repeat testing, particularly eliciting concerns regarding the window period. As with change talk regarding substance use, counselors are trained to reflect selectively and affirm statements favoring repeat testing.

PrEP referrals are discussed in Step 10. Participants are asked what they know about PrEP, and counselors provide additional information and/or referrals to PrEP providers.

In Step 11, counselors tie the goals and strengths from Component 1 into encouraging repeat testing and PrEP evaluation. Possible barriers to repeat testing and PrEP evaluation are discussed along with strategies to overcome these barriers.

Step 12 summarizes what was talked about during Component 2 while affirming the strengths and goals from Component 1. Here, counselors elicit goals with regard to repeat testing and/or PrEP uptake and elicit steps to achieve these goals that are achievable, clear, and have a distinguishable end point. Barriers to achieving the goal are elicited and problem-solved, and strengths are affirmed as a means of supporting follow-through with the goals established. Counselors then thank the participants for their time and end the session. Alternatively, if participants’ HIV test results are preliminary reactive, linkage to HIV care is encouraged in Steps 8 through 11. Step 8 focuses on participants’ reactions to the test result. Counselors allow the participants to process their emotions and use empathic reflections in response and offering statements of hope.

**Table 2:** Steps taken during Component 2 of the Swerve intervention.

Steps	Swerve intervention (SUBI^a^) Component 2a: Repeat HIV testing and PrEP^b^ (nonreactive results)	CTR^c^: HIV test counseling and partner services (nonreactive results)	Swerve intervention (SUBI) Component 2b: Linkage to HIV care (reactive results)	CTR: HIV test counseling and partner services (reactive results)
8	Response to testing nonreactive result	Meaning of test results	Response to testing HIV positive	Meaning of test results
9	Focus on repeat HIV testing	Cost and benefit analysis of testing	Focus on linkage to HIV care	Cost and benefit analysis of testing
10	PrEP referral	Interpretation of HIV test results	Links to Component 1 (goals, strengths, and substance use as a barrier)	Interpretation of HIV test results
11	Links to Component 1 (goals, strengths, and substance use as a barrier)	Reinforce plan for reducing risk based on test results	Summary and plan for action	Renegotiate risk reduction plan
12	Summary and plan for action	—	—	Discuss disclosure, partner services, appropriate referrals for medical evaluations, and early intervention services
13	—	—	—	Collect specimen for confirmatory testing^d^

^a^SUBI: substance use brief intervention.

^b^PrEP: pre-exposure prophylaxis.

^c^CTR: counseling, testing, and referral.

^d^Each ASO has specific procedure for confirmatory testing. Detroit-Blood draw for confirmatory testing: results in 3 days. Ypsilanti-Rapid test for confirmatory testing: results in 1 min. Flint-Rapid test for confirmatory testing: results in 20 min.

With permission, counselors begin to discuss the importance of linkage to HIV care in Step 9. Counselors explain how people with HIV can live healthy lives provided that they attend medical appointments and take medications while also exploring the participant’s perceived benefits of seeing a HIV medical provider.

Step 10 links the strengths and goals from Component 1 as a tool to continue to encourage linkage to care. Possible barriers to linkage to care are explored, with an emphasis on the potential impact of substance use.

Step 11 reflects on the participants’ goals for next steps toward linkage to care with an emphasis on eliciting goal-setting with goals that are achievable, clear, and have a defined end point. Counselors provide support to participants by affirming their strengths to meet these goals. Counselors thank the participants for their time and end the session.

#### Substance Use Brief Intervention at Study Visit 2

For those who received a nonreactive HIV test result at visit 1 and who are randomized to receive SUBI at study visit 2, the same intervention components and steps are delivered, as described above. For those who received a reactive test result at visit 1, the SUBI session focuses employing MI skills to address adherence with HIV care and the role of substance use.

### Sample Size and Power

The primary outcome for the proposed trial is successful engagement in care. For those who test seronegative at baseline, we define engagement in care as participation in routine HIV testing. For those who test seropositive at baseline, we define engagement in HIV care as linkage and retention in HIV care (per the Institute of Medicine guidelines of linkage within 30 days of diagnosis and at least 2 physician visits with a CD4 and viral load test in 12 months) and achievement of viral suppression. We define power as correctly identifying the difference in the proportions of YMSM with serospecific engagement in HIV care within 15 months of each active treatment condition (3 arms: SUBI-CTR, SUBI-SUBI, CTR-SUBI) to the control arm (CTR-CTR), thus powering for 3 independent hypothesis tests. Our sample size calculations are based on a 2-sample test of proportions using a 2-sided significance level of *P*<.05 altered by the number of comparisons using a Bonferroni adjustment (significance level is *P*=.02 for 3 comparisons). To have 80% power to compare each active treatment to the control group in a 4-arm trial, we require at least 500 participants to find a 20% difference between each treatment and control and 228 participants to find a 30% difference. To allow for 20% loss to follow-up (our previous trials have each achieved retention rates of >90%), we estimate a sample of 600 YMSMTG to be enrolled.

Participants may continue the study even if they miss follow-ups or visits intermittently over the data collection period. We will compare those who completed different follow-up interviews with those who did not on key predictors from the baseline assessment to check for possible sampling bias due to missing data. Missing data will be minimized by the computer-based entry for all measures. The use of Expectation-Maximization algorithm and multiple imputation approach in longitudinal analyses will help overcome missing data concerns when appropriate.

### Outcomes

The trial focuses on 3 sets of outcomes: engagement in HIV prevention and care services; AOD use; and sexual risk. The trial will also measure satisfaction with the intervention.

#### Engagement in HIV Prevention and Care Services

We will ask YMSMTG having unknown or negative HIV status to indicate the date of any recent HIV and STI tests. At baseline, we will ask participants to note if they have ever been medically diagnosed as having one or more STIs. Among those diagnosed, we will ask if the STI diagnosis occurred in the prior 12 months. In follow-up surveys, we will ask participants to report if they tested for STIs in the prior 3 months and whether any of their tests were reactive.

At each follow-up session (visits 3-7), we will measure the primary outcome of repeat HIV testing. For anyone who tested in each intersurvey period, we will assess the test result and motivations for testing (exposure-related vs regular health checking). For seropositives, the survey will assess the incidence of HIV-related physician visits, including whether CD4 counts and viral load tests were conducted and prescriptions for antiretroviral therapy (ART) were given. We will also ask participants to self-report their adherence to ART using an abbreviated 6-item questionnaire based on the AIDS Clinical Trial Group assessment. Participants are asked to note, using a scale ranging from “never” to “often,” if they missed their HIV medication over the past month for one of the listed reasons. Seropositives will also provide a blood sample for a viral load test. Viral load testing will be done every 6 months. We prioritize viral load tests over a biomarker of adherence, given that adherence is the primary pathway to viral suppression.

#### Biomarkers for HIV Prevention and Care

In addition to self-reported behavioral measures, the study will also collect biomarkers to test for HIV and STIs. At the intervention visits, HIV testing will be performed as part of CTR. STI screening: For syphilis screening, participants will undergo a blood draw for subsequent unheated serum regain test and, for chlamydia and gonorrhea screening, participants will provide a urine sample and pharyngeal, rectal, and/or vaginal swab cultures. Hepatitis C will be assessed using the OraQuick rapid test. Although we expect prevalence of hepatitis C to be low in an AOD population that has low levels of injection drug use (and thus hepatitis C will not be a secondary outcome), we will include hepatitis C testing to assess prevalence in this population. Those who are asymptomatic and test positive will be referred by study staff to local health care providers for further evaluation and treatment. All other STI tests will be processed by the Michigan Department of Health and Human Services. As required by state law, all new positive STI results will be reported to the State Health Department for the purposes of disease surveillance. The requirement for reporting of confidential test results to the health department will be explained in the informed consent. Condoms and water-based lubricant will be provided to all participants. We will screen for STIs at the final study assessment.

#### Alcohol and Other Drugs Use, Misuse, and Consequences

We use the ASSIST [49] to assess frequency of AOD use in prior 3 months, a validated measure to screen for the presence of possible alcohol and other substance use disorders. For each substance, the ASSIST assesses frequency of use, cravings, impact of use on key life domains, expressed concern from others, and failed quit attempts to derive a Specific Substance Involvement Score. If respondents indicate alcohol use, we ask the respondent’s frequency with which they had had 5 or more drinks in a row during the last 2 weeks (binge drinking), and how often the respondent drinks to get drunk. We also assess respondents’ use of alcohol and/or illicit drugs during or before condomless sex. We use the Alcohol Use Disorders Identification Test [55], a 10-item screening questionnaire with 3 questions on the amount and frequency of drinking, 3 questions on alcohol dependence, and 4 on problems caused by alcohol.

#### Biomarkers for Use of Alcohol and Other Drugs

In addition to self-reported behavioral measures of drug and alcohol use, the study will also collect biomarkers of drug and alcohol use. Participants will provide urine samples for on-site toxicology screening using an EZ split key test kit (Redwood Toxicology Laboratory, Inc.). In our prior clinical research with methamphetamine-using MSM, urine samples were obtained for toxicology testing in 98% of study visits. The on-site urine screening kits are designed to test for recent amphetamine, barbiturates, benzodiazepines, buprenorphine, cocaine, marijuana, methadone, methamphetamine, oxycodone, propoxyphene, morphine, and ecstasy. Urine toxicology screening will be used to provide information regarding recent substance use and will be conducted each study visit. Alcohol screening will be used where ethyl glucuronide (EtG) a direct metabolite of ethanol indicates that ethanol has been ingested within the last 3-4 days (80 hours). EtG will be analyzed using the 1-step EtG test dip card. Urine will be collected in a urine specimen cup and the tip of the EtG dip card will be submerged into the urine sample for 15 seconds. EtG will allow us to adjust for under-reporting of recent unhealthy drinking on participants’ surveys and will also be screened at each study visit.

#### Sexual Behaviors

Sexual behaviors will be assessed using a modified version of the Sexual Practices Assessment Schedule (SPAS) used in previous studies with YMSMTG [56] to explore different sexual acts (oral, vaginal, and anal) with different partner types. SPAS estimates the number of sexual partners and occasions across partner types, as well as the proportion of instances when condoms were not used. We also assess how frequently they report using drugs or alcohol immediately before or during sex. SPAS also ascertains YMSMTG’s HIV status disclosure practices with each partner.

#### Intervention Acceptability and Satisfaction

YMSMTG will report data on the acceptability of the intervention after completing each intervention session. We will use 2 different assessments: (1) Self-Evaluation Forms (SEF) and (2) Client Satisfaction Questionnaires (CSQ-8). The SEF is a brief 12-item questionnaire that elicits information about the experience with the intervention (ie, was the intervention interesting, was it relevant to their life, and did they learn from the intervention). The CSQ-8 will be used at the completion of the intervention to assess satisfaction with the intervention. The CSQ-8 has demonstrated high internal consistency across a large number of studies [57]. The SEF and CSQ will take approximately 10 min to complete and will be completed at the ASO on a tablet immediately after intervention delivery at visits 1 and 2.

### Statistical Analysis

Descriptive statistics of the clinical and demographic characteristics of the participants will described for all and by treatment group. These will be compared between treatment groups using *t* tests or Wilcoxon rank sum tests for continuous variables and chi-square tests for categorical variables. We will conduct primary analyses of our pooled successful engagement measure using logistic regression analyses to compare each active treatment group with the control in pairwise comparison tests at an adjusted significance level of .017 to reduce Type-I errors in our 4-arm trial. We will then conduct exploratory logistic regression analyses by sero-status. For seronegative YMSMTG, the proportion of participants who obtain at least 2 tests at least 3 months apart within 15 months will be calculated and presented with corresponding 95% exact binomial CIs. Among seropositive YMSMTG, we will examine how intervention conditions influence HIV linkage and retention in care per IOM guidelines. We will also examine for all participants the pursuit of substance use treatment services (if necessary). Hence, for seronegative participants, the outcome will be repeat HIV testing at regular intervals, and for seropositive participants, the outcome will be linkage and retention in care. We will not consider viral suppression in the regression analysis due to the short time period available to achieve suppression. However, we will present descriptive statistics on viral load and suppression across the 4 treatment arms. The regression will be run with group assignment in the model while controlling for patient, structural, and agency characteristics. Interactions between group assignment and these characteristics will be tested to explore potential moderators of treatment effect. We will repeat these analyses for STI/ATOD biomarkers conducted during the trial. Additional analyses include comparing groups in (1) the average number of tests obtained using Poisson regression and (2) time to getting tested using repeated events survival analysis.

We will test for intervention effects over time on sexual risk (eg, CAI events) and drug use (eg, reduction in ATOD use and ATOD-related consequences) outcomes. We will use the general framework of generalized linear mixed models [58-60] to model the longitudinal outcome trajectories [61-63]. Note that some of our outcomes are measured as binary, some as count, and some as continuous measures and thus need to be treated differently. Assuming a linear time trend, visit can be coded from 0 to 7, or it can be simply coded as a categorical variable representing the distinct effect of each visit compared with the baseline. The interaction coefficients are of interest here, measuring the difference in the rate of change in outcomes across the 4 treatment groups. The subject-specific random intercepts *β*_0i_ are assumed to be normally distributed with a common variance and they account for within-person correlation. We will also explore if we need a subject-specific random slope corresponding to visit in the above model. Maximum likelihood estimation will be used for fixed effect parameters. To ensure robustness, we will also apply an exchangeable working correlation structure to its corresponding generalized estimating equation model [64].

### Intervention Fidelity and Supervision

The study team balanced the clinical and ecological validity of the design and procedures of project Swerve as the intervention was developed for YMSMTG in the DMA. First, we recognize that the CTR and SUBI treatments may have different amounts of contact time with participants. Although it is possible that contact time may influence intervention effects across CTR and SUBI conditions, the control sessions may actually last longer than often expected during CTR, given the high-risk characteristics of our study participants. In our analyses, we will also examine whether length of sessions differ statistically across treatment arms and include time spent in each session as a covariate due to its potential confounder. Second, we recognize that there may be variability in how counselors deliver CTR and SUBI sessions, which could confound our ability to measure the intervention’s strength. We have put in place several procedures to minimize potential biases including training counselors using 2-day training with boosters and ongoing group and individual supervision, allowing counselors at each site—who are not trained in the intervention—to deliver CTR, and monitoring and addressing treatment fidelity for both conditions throughout the trial using the Motivational Interviewing Treatment Integrity-4 rating system [65].

### Examine Cost-Effectiveness and Sustainability

The study team will collect information on: (1) time spent by study staff for training, supervision, and technical assistance of counselors; (2) time participants spent in a counseling session; and (3) costs associated with test counselor delivery of the intervention. Capital equipment cost (eg, computer) and facility cost (eg, rent, telephone) at the study sites that are attributable to our intervention will be obtained from each site’s accounting records. Capital equipment cost will be distributed over a 4-year period with a 3% discount rate. Cost items that are not directly divisible between participants will be spread across relevant individuals (eg, spreading capital equipment and facility cost across all participants tested at each agency). A flat rate covering the cost of CTR materials for each intervention group will be estimated. No costs associated with research data collection will be included. These components of cost will be summed over the 15-month study period for each participant to generate an estimated per person cost. Effectiveness will be measured by examining relevant substance use and HIV-related outcomes reported by each YMSMTG over the 15-month period. Incremental cost effectiveness ratio (ICER) across treatment arms will be defined as ΔC/ΔE, where ΔC denotes the estimated difference in mean cost per intervention and ΔE reflects the estimated difference in mean effectiveness between the intervention and control group. ICER indicates the additional costs associated with the intervention for each new HIV infection avoided. Nonparametric bootstrap resampling will be used to estimate the 95% CI of ICER [63]. Primary analysis will be performed on participants with complete data. Sensitivity analysis will be conducted by including all participants with multiple imputation for those with missing data.

## Results

Project Swerve launched in April 2017 and is currently recruiting YMSMTG into the trial. Current recruitment strategies combine online and in-person (venue-based sampling) approaches. As of February 22, 2018, 5183 people began the screener survey, of which 3178 (61.32%) completed it. In total, 594 (18.70%), people successfully screened were eligible to participate in the study, of which 378 (63.6%) provided consent and 223 of these (58.9%) enrolled into the study. Of these, 160 (71.7%) have completed the baseline survey and 18 dropped from the study; the remaining 142 participants have been randomized into study arms as follows: 36 SUBI-SUBI; 36 SUBI-CTR; 34 CTR-SUBI; and 36 CTR-CTR.

## Discussion

SUBI is a promising approach to address AOD use as part of HIV prevention and care services for YMSMTG. Efficacious SUBI approaches are typically delivered on-site to clients and have the advantage of being reimbursable [66] and cost-effective [67]. For nontreatment-seeking samples, SUBI has strong support in the alcohol literature [68-74], and some promising effects have been observed with respect to other substances, including heroin, cocaine, amphetamine, and marijuana use [68-73,75]. Few SUBI trials have considered whether the dosage or sequence of SUBI may result in differential risk-reduction outcomes, particularly among youth. In a 2012 meta-analysis, Eaton and colleagues [74] showed that one-time, brief interventions were a suitable and efficacious strategy for HIV and STI prevention. Pooling together 29 intervention trials (n=52,465), the authors found that single-session interventions (1) were associated with a reduction in STI incidence and risk behaviors when compared with standard-of-care; (2) were as effective as multisession interventions; (3) and were particularly effective in trials involving racial and ethnic minorities. In a recent study among drug-using adults in the DMA, Bonar and colleagues [50] found that a brief MI-focused intervention targeting drug use resulted in postintervention changes in psychological precursors of drug use behavior change (eg, confidence and intentions to reduce drug use), reduced drug use, and increased intentions to use condoms with sexual partners.

At present, few RCTs have examined the efficacy of brief interventions targeting ATOD use as a strategy to reduce sexual-risk taking behavior or to increase engagement in HIV care and prevention among high-risk YMSMTG who, compared with heterosexual adults or older MSM, may not have yet developed sustained drug use and abuse patterns. Furthermore, distinct developmental considerations including the use of ATOD before the legal age for use, the high prevalence and visibility of ATOD within YMSMTG’s social networks, and the influence of these social networks’ norms on YMSMTG’s behavior may require particular attention when developing a SUBI for YMSMTG [76-80]. In addition to targeting an often overlooked barrier to successful engagement in HIV prevention and care, our intervention may offer structural opportunities to offset the decreases in HIV prevention funds across ASOs in the region. In light of shrinking HIV prevention and care funds, the Swerve program could increase ASO revenue by billing for substance use screening and referrals. Consequently, if efficacious, our theoretically-guided intervention may provide HIV and substance use risk reduction strategies that recognize the developmental needs specific to YMSMTG.

## Abbreviations

AOD: alcohol and other drugs
ASO: AIDS Service Organization
CAI: condomless anal intercourse
CDC: Centers for Disease Control and Prevention
CSQ: Client Satisfaction Questionnaires
CTR: counseling, testing, and referral
DMA: Detroit Metro Area
ICER: incremental cost effectiveness ratio
MI: motivational interviewing
MSM: men who have sex with men
PrEP: pre-exposure prophylaxis
RCT: randomized controlled trial
SUBI: substance use brief intervention
SUDs: substance use disorders
SEF: Self-Evaluation Forms
STI: sexually transmitted infections
TG: transgender people
YMSM: young men who have sex with men
YMSMTG: young men who have sex with men and transgender people
